# The Role of Opioid Receptors in Immune System Function

**DOI:** 10.3389/fimmu.2019.02904

**Published:** 2019-12-20

**Authors:** Toby K. Eisenstein

**Affiliations:** Center for Substance Abuse Research, Lewis Katz School of Medicine at Temple University, Philadelphia, PA, United States

**Keywords:** opioids, immunosuppression, infection, sepsis, chemokines, cytokines, toll-like receptors

## Abstract

Research on the effects of opioids on immune responses was stimulated in the 1980s by the intersection of use of intravenous heroin and HIV infection, to determine if opioids were enhancing HIV progression. The majority of experiments administering opioid alkaloids (morphine and heroin) *in vivo*, or adding these drugs to cell cultures *in vitro*, showed that they were immunosuppressive. Immunosuppression was reported as down-regulation: of Natural Killer cell activity; of responses of T and B cells to mitogens; of antibody formation *in vivo* and *in vitro*; of depression of phagocytic and microbicidal activity of neutrophils and macrophages; of cytokine and chemokine production by macrophages, microglia, and astrocytes; by sensitization to various infections using animal models; and by enhanced replication of HIV *in vitro*. The specificity of the receptor involved in the immunosuppression was shown to be the mu opioid receptor (MOR) by using pharmacological antagonists and mice genetically deficient in MOR. Beginning with a paper published in 2005, evidence was presented that morphine is immune-stimulating via binding to MD2, a molecule associated with Toll-like Receptor 4 (TLR4), the receptor for bacterial lipopolysaccharide (LPS). This concept was pursued to implicate inflammation as a mechanism for the psychoactive effects of the opioid. This review considers the validity of this hypothesis and concludes that it is hard to sustain. The experiments demonstrating immunosuppression were carried out *in vivo* in rodent strains with normal levels of TLR4, or involved use of cells taken from animals that were wild-type for expression of TLR4. Since engagement of TLR4 is universally accepted to result in immune activation by up-regulation of NF-κB, if morphine were binding to TLR4, it would be predicted that opioids would have been found to be pro-inflammatory, which they were not. Further, morphine is immunosuppressive in mice with a defective TLR4 receptor. Morphine and morphine withdrawal have been shown to permit leakage of Gram-negative bacteria and LPS from the intestinal lumen. LPS is the major ligand for TLR4. It is proposed that an occult variable in experiments where morphine is being proposed to activate TLR4 is actually underlying sepsis induced by the opioid.

## Introduction

Beginning in 1975, a robust literature has accumulated to support the conclusion that opioids modulate immune responses. Overwhelmingly, these studies have shown that opioids are immunosuppressive. However, with the publication of a paper in 2005, the hypothesis was formulated that opioids are pro-inflammatory, and that the resulting opioid-induced inflammation mediates addiction. This paper will critically review the evidence that opioids affect functioning of the immune system, and whether the result of this interaction is immunosuppressive or immuno-stimulating. The review will also address mechanisms by which opioids alter immune function, specifically whether there is a direct effect of these drugs on cells of the immune system, or whether the effects are mediated through activation of the hypothalamic-pituitary-adrenal (HPA) axis, the sympathetic nervous system, or other pathways. These studies are of relevance to public health, as there was, and still is, a strong intersection between intravenous drug use and HIV incidence. A major question in this arena has been whether opioids, through their immunomodulatory effects alter host defense to the virus.

## Early Discovery That Opioids Are Immunosuppressive

**Figure 1:**
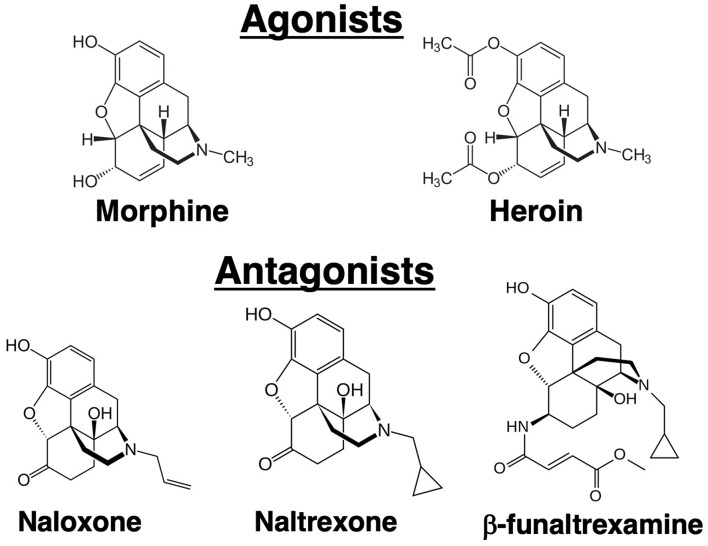
Structures of alkaloid agonists and antagonists.

In 1979, a paper was published in the Journal of Immunology by Wybran et al., which had a major impact on our understanding of the physiological effect of opioids on the immune system (1). It was reported that morphine inhibited the rosetting of human peripheral blood T cells with sheep red blood cells (SRBCs). Prior to wide-spread use of flow cytometry, the fortuitous ability of SRBCs to adhere to human T cells, but not B cells, was used as the technique for distinguishing the numbers of these two cell populations in a blood sample. What Wybran observed was that the morphine-induced inhibition of rosetting could be blocked by pretreatment with naloxone, an antagonist at opioid receptors (Figure 1). Further, it was shown that the endogenous opioid peptide, met-enkephalin, enhanced the red blood cell rosetting, a phenomenon which was also blocked by naloxone. The Wybran paper set the stage for our future understanding of a broad class of natural interactions between the neural and immune systems, as aptly described in this quote from the paper, “The findings of receptors on T lymphocytes for drugs and substances known to bind to nervous cells may provide further insight into the relations between the immune system and the central nervous system (CNS). Such links may be of utmost importance in various disease states like slow virus infection, multiple sclerosis, and perhaps neurotic and psychotic disorders where immune mechanisms may be involved.” On the larger scale, these words have come to be prophetic. Of immediate interest was the implication of these observations, namely that leukocytes express opioid receptors. In 1982 the AIDS (Acquired Immunodeficiency Syndrome) epidemic emerged. It was recognized that patients suffered from a severe depression in T cell numbers, but the viral cause was not identified until 1986. At the beginning of the outbreak, epidemiologic evidence showed that one third of AIDS patients were intravenous drug abusers, mainly of heroin (2). Based on the Wybran paper, the question arose as to whether the opioids were in some way mediating the immune suppression observed in AIDS patients. The National Institute on Drug Abuse introduced an initiative to fund research into the effect of opioids on the immune system. The papers relating to effects of opioids on immune responses are summarized in Table 1.

**Table 1:** Effects of opioids on immune functions.

**Function**	**Species**	**References**
Suppression of natural killer cell activity	Mouse *in vivo* Rat *in vivo* Human *in vivo*	(20)^*^, (21) (10–18) (22)
Suppression of cellular responses to mitogens *ex vivo*	Mouse Rat Human	(20)^*^, (23, 25) (26–28) (30)
Depression of antibody production	Mouse *in vivo*	(21, 23, 31, 33–35)^**^, (36–38, 40)
	Mouse *in vitro*	(42)^***^, (43)
Depression of T cell mediated adaptive immune responses	Mouse *in vivo*	(44, 45)
Depression of cellularity	Mouse *in vivo*	(21, 23, 47)
Induction of apoptosis	Mouse *in vivo*	(50)
	Mouse *in vitro*	(50)
	Human *in vitro*	(48, 49)
Inhibition of cell growth	Mouse *in vivo*	(15, 53, 55)
	Mouse *in vitro*	(20, 51)
	Human *in vitro*	(52, 56)
	Monkey *in vivo*	(54)
Suppression of phagocytosis	Mouse *in vivo* Mouse *in vitro*	(58, 59) (60–64)
Down-regulation of cytokines and other inflammatory associated mediators	Mouse *in vivo* Mouse *in vitro* Rats *in vivo* Human *in vitro*	(20, 84–86) (34, 75, 79–81, 83, 88) (73, 74, 78, 89) (67, 69–72, 82)

* *Also used hydromorphone, codeine and oxycodone*.

** *Also used U50,488H and deltorphin II*.

*** *Used DAMGO*.

## Opioid Pharmacology as Background

Opioid receptors in the brain were initially discovered using biochemical techniques that demonstrated binding of radiolabeled opioid ligands. Three major receptors were discovered that were designated mu, kappa and delta (3–5). In 1993, these receptors were cloned (6–8). Currently, they are termed mu opioid receptor (MOR), kappa opioid receptor (KOR), and delta opioid receptor (DOR). The endogenous ligands for these receptors are the neuropeptides: β-endorphin (MOR), dynorphin (KOR), and methionine-enkephalin (DOR), although these proteins have significant cross-affinity for the other receptors. Morphine has greatest affinity for the MOR, but it can bind to a lesser degree to the other receptors. The analgesic and psychoactive effects of morphine, as well as the adverse effects of respiratory depression and inhibition of gastric transit, are mediated through the MOR. Unlike the endogenous peptide ligands for the opioid receptors, morphine is an alkaloid (Figure 1), a natural product extracted from the opium poppy. Heroin is synthetic diacetyl-morphine. Whereas, intravenous drug abusers at the time of the AIDS epidemic were mostly injecting heroin, subsequent laboratory studies have mostly employed morphine as the opioid. It is felt that results from morphine are generalizable to heroin because heroin is metabolized by deacetylation to morphine. Practical considerations also underlay decisions to use morphine in the laboratory because it is less lipophilic than heroin, making it easier to dissolve, and it is a Schedule 2 rather than a Schedule 1 drug, making it easier to obtain. Results from morphine were also judged to have wider applicability, as morphine, but not heroin, is used therapeutically in millions of patients. Since the MOR is the major target of both compounds, results obtained with morphine are believed to be directly translatable to heroin abuse, although there is some evidence for unique biologically active heroin metabolites (9). Naloxone and naltrexone are major antagonists of the opioid receptors (Figure 1). Both synthetic compounds bind to all three opioid receptors. Some investigators have also used β-funaltrexamine (β-FNA), another antagonist, which is selective for MOR. Narcan® is the brand name for commercially marketed naloxone. It has greater affinity for the MOR than morphine, so it is able to displace morphine from the MOR without inducing receptor signaling. A difficulty in working with morphine is that after only short exposures animals can develop physical dependence to the drug. As the half-life of the drug in mice and rats, as in humans, is in the range of several hours, when it is desired to test the effect of more than a single, acute exposure to the drug, strategies are needed to administer morphine without inducing withdrawal. Withdrawal has myriad physiological effects which certainly could affect immune responses. Approaches to maintain chronic levels of morphine include multiple injections over the course of a day, use of slow-release morphine pellets, or use of osmotic mini-pumps. Slow-release pellets are formulated using a nitrocellulose matrix which dispenses the drug slowly over 7 days. Placebo pellets containing only the matrix, and naltrexone pellets releasing the antagonist, are also available. Pellets are implanted surgically, subcutaneously in a skin pocket beside the spine in a manner similar that used to implant osmotic pumps. A further complication with using morphine and similar opioids is that continued exposure leads to “tolerance” to the analgesic effects of the drugs. Like immunological tolerance, opioid tolerance means that that animal no longer responds to the drug or has a lesser response to a given dose of the opioid. Interestingly, tolerance does not develop to the constipating effects of opioids. A question which has been investigated is whether there is tolerance to immunomodulatory effects of opioids, and this topic is considered later in this review.

## Opioids and Suppression of Natural Killer (NK) Cell Activity

Among the earliest studies testing the effect of opioids on immune function were those that examined NK cell activity, a measure of innate immunity. Shavit et al. showed that morphine given to rats by subcutaneous (s.c.) injection for 4 days suppressed NK cell activity in the spleen, and that N-methylmorphine, which does not pass the blood-brain barrier, was inactive (10–12). The latter observation suggested that the effect of morphine was mediated through the neuronal system, rather than by acting directly on immune cells in the periphery. This conclusion was reinforced by a high-profile paper in Science by Weber and Pert which showed that injecting morphine directly into the periaqueductal gray region of the rat brain (which is involved in pain sensing) depressed the NK cell activity in spleen 3 h later (13). Similar injections into 5 other brain regions were without effect. Naltrexone blocked the NK suppressive activity of morphine in both studies. Evidence for involvement of adrenergic and sympathetic neurotransmitters, of glucocorticoids, and of dopaminergic and Peptide Y signaling as mediators of opioid immunosuppression of NK cell activity have been found (14–18). Franchi et al. found that subcutaneous injection of rats with 2 doses of morphine, but, interestingly, not buprenorphine, spaced 5 h apart, reduced splenic NK cell activity (19). Sacerdote et al. reported that morphine given *in vivo* inhibited NK cell activity of mouse spleen cells *ex vivo* (20). Further proof that opioid receptors mediate the suppression of NK cells was provided by Gaveriaux-Ruff who found that MOR knock-out (k/o) mice did not respond to morphine with a decrease in NK cell activity (21). Interestingly, studies have also been carried out in humans to test the effect of morphine on NK cell activity. Yeager et al. administered morphine intravenously for 24 h to normal, non-opioid abusing volunteers in the hospital, and obtained NK cells from peripheral blood by venipuncture before administration of the opioid, and 2 and 24 h later. Morphine administration resulted in a significant depression in NK cell activity at both time points compared to baseline (22). The studies cited above support the conclusion that morphine suppresses NK cell activity in rats, mice and humans, and that the mechanism of the immunosuppression is through the MOR. However, for suppression of NK cell cytotoxicity the effect of morphine does not appear to be direct, but rather is mediated by signals from the neural system.

## Opioids and Suppression of Responses to Mitogens

An early observation about the effect of opioids on immune responses was published from the laboratory of Holaday showing that morphine pellet implantation inhibited the response of mouse spleen cells *ex vivo* to the T cell mitogen, Concanavalin A (ConA), and to the B cell mitogen, bacterial lipopolysaccharide (LPS) (23). These effects were not evident in mice treated with RU486, an inhibitor of glucocorticoids, or in adrenalectomized mice (24). Thomas et al. (25) also reported that morphine depressed B cell proliferation stimulated by anti-IgM and IL-4. Bayer's group reported that peripheral blood T cells, harvested 2 h after a subcutaneous (s.c.) injection of rats with morphine, were markedly suppressed in their response to ConA (26). The immunosuppressive effects were not duplicated by N-methyl-morphine, leading to the conclusion that central opioid pathways were involved (27). In contrast to the findings of Holaday using mouse spleen cells from animals implanted with a slow-release pellet, the immunosuppression of rat peripheral blood cells to ConA, induced by a single, acute injection of morphine, was not abolished by adrenalectomy, hypophysectomy, or administration of the glucocorticoid antagonist, RU486 (28). Chlorisondamine, a ganglionic blocker, did inhibit the immunosuppression (29). Govitrapong et al. tested the responses of T cells to phytohemagglutinin (PHA) in peripheral blood of heroin addicts and in addicts in withdrawal from the opioid. In both cases, T cell responses were depressed for up to 2 years (30). Thus, opioids were shown to suppress mitogen responses of T cells in mice, rats, and humans, and of B cells in mice when drugs were given *in vivo*. Sacerdote et al. reported that morphine, but not hydromorphone, codeine, or oxycodone inhibited the mitogen responses of normal mouse spleen cells when the drugs were given *in vivo* and spleen cells were tested ex *vivo* (20).

## Opioids and Suppression of Antibody Production

### Opioids Given *in vivo* and Immunosuppression

The first paper showing that morphine inhibited antibody responses by mouse spleen cells to SRBCs as the antigen was published in 1975 (31). High doses of morphine (75 mg/kg) were injected one day before injection of SRBCs and for 3 days thereafter. Splenic cells from treated or placebo animals plated *in vitro* and incubated with an excess of SRBCs and complement revealed the number of B cells secreting antibody to the SRBCs, which in the presence of complement lysed the SRBCs producing visible plaques in the lawn of red blood cells. This method is called the plaque-forming cell (PFC) assay and measures the number of cells secreting IgM anti-SRBC antibodies (32). Bussiere et al. administered morphine to mice using slow-release pellets and also found that spleen cells placed *ex vivo* 72 h after pellet implantation had markedly depressed PFC responses compared to placebo pelleted animals (33). Simultaneous implantation of a naltrexone pellet with a morphine pellet blocked the immunosuppressive effect of morphine, and naltrexone alone had no effect. Kinetic experiments showed that after morphine pellet implantation, onset of suppression of the PFC antibody response was gradual, reached a maximum at 48 h and dissipated by 120 h, which was interpreted as development of tolerance to the immunosuppression (34). Bryant et al. had found a similar time course for morphine-induced suppression of mitogen responses (23). Minipumps have also been used to dispense opioids selective for MOR, KOR, or DOR to test the effect of different opioid ligands on PFC responses (35). This mechanism of dispensing the drugs allowed testing of agonists for which there are no pellets, and also for carrying out dose-response studies. It was found that morphine sulfate (primarily a mu agonist), U50,488H (a kappa selective agonist) and deltorphin II (a delta_2_ selective agonist) dispensed for 48 h, each inhibited the *ex vivo* PFC responses of spleen cells, yielding U-shaped dose-response curves. Co-implantation of mini-pumps dispensing antagonists that were receptor selective (CTAP vs. morphine; nor-binaltrophimine vs. kappa, and naltriben vs. delta_2_) blocked the immunosuppressive effects of the agonists (35). Morphine pellet administration has also been shown to inhibit secretory IgA responses to cholera toxin in gastrointestinal lavage fluid or produced by *ex vivo* ileal organ cultures from morphine treated mice (36, 37). Morphine pellets have also been shown to decrease the serum immune response to tetanus toxoid when this antigen was given 72 h post pellet implantation in mice, as assayed quantitatively by ELISA (38). Morphine pellets also suppressed the murine serum antibody titer to trinitrophenylated bovine serum albumin, which was blocked by naltrexone (39). Intravenous morphine administered to rats immediately after injection of keyhole limpet hemocyanin depressed serum antibody responses to this antigen and the effect was inhibited by naltrexone (40). The experiments cited above on antibody responses involved administration of morphine *in vivo* and assessment of antibody responses *in vivo* or *ex vivo*. They all support an immunosuppressive consequence of morphine exposure. The Kieffer laboratory definitively showed that the MOR mediated the immunosuppressive effects of morphine by testing NK cell activity, proliferative responses of lymphocytes to ConA and LPS, and immunoglobulin levels in LPS-stimulated culture supernatants in wild-type (WT) and MOR k/o mice (21). WT mice demonstrated immunosuppression to this panel of immune responses, but MOR k/o mice were not suppressed. A lingering question in the field as these results on opioid suppression of antibody responses emerged was whether the immunosuppression was due to a direct effect of the opioid on cells of the immune system or whether the effects were indirect through opioid-induced activation of other physiological systems. Pruett et al. argued that part of the suppression of the humoral immune response was due to activation of the HPA axis (41). As noted above in the section on opioids and NK cells, the sympathetic nervous system, the adrenergic system, dopaminergic pathways and Neuropeptide Y have all been implicated in mediating the effects of morphine. As a counter to this hypothesis is the definitive evidence that opioids added *in vitro* to immune cells suppress antibody responses *in vitro*.

### Opioids Applied *in vitro* and Immunosuppression of Antibody Responses

In these experiments, spleen cells were taken from opioid naïve mice and were placed in culture where experimental wells received an opioid and control wells received medium. Other neurotransmitter systems of the body cannot be involved in immunosuppression observed by adding opioids to purified cells of the immune system *in vitro*. Taub et al. reported that addition of DAMGO, a synthetic peptide that is a selective agonist at the MOR, or U50,488H, that is a kappa selective agonist, resulted in inhibition of the PFC response of mouse spleen cells to SRBCs (42). The inhibition of antibody induction was blocked with naloxone, and also with a kappa selective antagonist, respectively. Eisenstein et al. reported that morphine also produced a dose-dependent inhibition of the PFC response which was naloxone reversible and was mouse strain dependent (43).

Thus, the evidence indicates that there are both direct effects of opioids on immune cells, as well as mediated, indirect effects.

## Opioids and Depression of T Cell Mediated Adaptive Immune Responses

Several laboratories have reported that morphine administration *in vivo* blocks adaptive T cell responses. These include reports by Bryant and Roudebusch showing that morphine pellets depressed development of contact sensitivity to picryl chloride and inhibited a graft vs. host reaction in mice (44). Dafny found that morphine blocked development of delayed-type hypersensitivity in rats to attenuated mycobacteria (45), and Molitor et al. reported inhibition of sensitization to 2,4-dinitrofluorobenzene in pigs (46).

## Morphine and Depression of Cellularity, Induction of Apoptosis, and Inhibition of Cell Growth

Early studies using morphine slow-release pellets showed that drug administration resulted in splenic and thymus cell atrophy (23, 47). Later investigation of this phenomenon provided evidence that morphine added to human peripheral blood mononuclear cells (PBMCs) (48) or to human monocytes (49) *in vitro* induced apoptosis, in the monocytes by induction of nitric oxide. In a study of morphine-induced apoptosis, Yin et al. confirmed that morphine administration to mice *in vivo* resulted in reduced cellularity in the spleen, and showed that the opioid induced Fas in the spleen, heart and lung (50). *In vitro* studies showed that morphine induced Fas in a T cell hybridoma and in human peripheral blood lymphocytes. Addition of Fas ligand (FasL) triggered apoptosis (50). In another line of investigation, Roy's laboratory reported that morphine added to murine bone marrow cultures inhibited formation of macrophage colonies in soft agar from precursors (51). Vassou et al. (52) found that morphine inhibited *in vitro* proliferation of human multiple myeloma cells (B cells). Several investigators have also examined effects of morphine pellets on lymphocytes. Altered ratios of CD4 and CD8 T cells were found in the spleen and thymus of mice (15, 53), as well as in monkeys that received daily injections of morphine for 2 years (54). Zhang et al. carried out a more detailed study of the effect of morphine pellets on lymphocyte subsets in the spleen and lymph nodes of mice 7 days after pellet implantation and found reduced numbers of B cells and CD4 and CD8 T cells (55). B cells that were most vulnerable to morphine depletion were IgM^+^IgD^−^. In the bone marrow, the number of B cell precursors was reduced. Naïve, memory, and effector memory CD4 and CD8 T cells were all depleted by morphine in the spleen and lymph nodes. Roy et al. showed that morphine added *in vitro* to human PBMCs or to mouse spleen cells stimulated with anti-CD3/anti-CD28 polarized the T cells to a Th2 phenotype, as evidenced by up-regulated production of IL-4 and IL-5 and decreased IL-2 and IFN-γ (56). Morphine treatment also increased NFAT binding to response elements in cellular DNA. Sacerdote et al. also reported that morphine down-regulated IL-2 production when added to Con A-stimulated mouse spleen cells *in vitro* (20). These studies provide evidence that morphine depresses numbers of major classes of lymphocytes when given *in vivo* or when added to cells *in vitro*. There is still controversy as to whether the *in vivo* effects are mediated by corticosteroids (57).

## Effects of Opioids on Cell Subsets and Cytokines

### Opioids Suppress Phagocytosis and Microbicidal Activity of Phagocytes and Enhance Viral Replication

An essential aspect of innate immunity is the ability of the neutrophils and macrophages to ingest and kill microbes. The literature on this subject overwhelmingly indicates that morphine suppresses phagocytosis and microbicidal activity of phagocytes. The first demonstration of this phenomenon was reported by Tubaro et al. who administered morphine to mice by s.c. injection for 3 days and then harvested elicited peritoneal macrophages that were tested *ex vivo* for capacity to engulf and kill the fungus, *Candida albicans*. It was shown that morphine depressed phagocytosis and fungicidal activity of macrophages which correlated with reduced production of superoxide anion (58). These results were confirmed in regard to inhibition of phagocytosis of *Candida albicans* using slow-release morphine pellets and harvesting unelicited peritoneal macrophages 48 h after pellet implantation which were tested for phagocytic capacity against the fungus (59). Naltrexone blocked the morphine-induced suppression. Importantly, in this study as well as many others, the effect of opioids added to phagocytic cells *in vitro* was tested, and it was found that phagocytosis and microbicidal activity were directly inhibited (59). Szabo et al. also used *Candida albicans* as the target and showed that compounds selective for mu, kappa and DORs all blocked ingestion of the fungus by mouse peritoneal macrophages treated with the opioid *in vitro*. Further, antagonists specific for these receptors inhibited the effect, definitively demonstrating that the opioids are directly acting on these phagocytic cell through opioid receptors (60). Wang et al. reported that murine alveolar macrophages obtained by broncho-alveolar lavage and treated with morphine *in vitro* had depressed phagocytosis and killing of *Streptococcus pneumoniae* (61). A series of papers from the laboratory of Reynaud demonstrated that morphine added *in vitro* to mouse peritoneal macrophages inhibited the Fc-mediated phagocytosis of sheep red blood coated with antibody (62). In a collaborative study with Roy and Loh, this system was probed with pharmacological precision carrying out dose response curves with selective mu, delta, and kappa agonists and antagonists (63). It was found that mu and delta_2_ agonists inhibited phagocytosis in a dose-dependent manner. Ninković and Roy, using the J774.1 macrophage cell line, provided a mechanism by which morphine added *in vitro* could disrupt phagocytosis by inhibiting actin polymerization through inhibition of Rac1-GTPase and p38 mitogen-activated protein kinase (MAPK) (64). Another aspect of the innate immune response is the ability to control viral infections. Peterson et al. reported that morphine added to human promonocytes cultured from human brain tissue exacerbated replication of HIV with an inverted U-shaped dose response curve (65). Reports from the laboratory of Rogers supported potentiation of HIV replication in monocytes by morphine (66). Ho et al. showed that heroin (67) and methadone (68) added to human macrophages *in vitro*, enhanced HIV replication, also with an inverted U-shaped dose response curve.

### Effect of Opioids on Macrophages, Microglia, and Macrophage/Microglial-Derived and T Cell-Derived Cytokines and Other Inflammatory Associated Mediators

Peterson and colleagues reported that in human PBMCs, morphine blocked production of the reactive oxygen intermediates, superoxide and peroxide, that are involved in microbicidal mechanisms of phagocytes in response to opsonized zymosan (69). Interferon-γ and TNF-α were also depressed by morphine (70, 71). Evidence was presented that the immunosuppressive cytokine, transforming growth factor-beta (TGF-β), produced by lymphocytes, was mediating the morphine-induced down-regulation of reactive oxygen intermediates (72). Roy's laboratory reported that morphine added to murine bone marrow cultures inhibited formation of macrophage colonies in soft agar from precursors (51). Bussiere et al. explored the mechanism of immunosuppression of *ex vivo* antibody formation by mouse spleen cells induced by morphine pellet implantation. Using co-cultures of normal spleen cells or fractionated spleen cell populations, they showed that addition of adherent, but not non-adherent spleen cells from normal animals restored the PFC responses of spleen cells taken from morphine-treated animals. Further, antibody responses could be restored by addition of cytokines produced by macrophages, IL-1β or IL-6, or by the macrophage-activating cytokine, IFN-γ. These results support the conclusion that morphine depressed either macrophage numbers or production of macrophage pro-inflammatory cytokines (34). Wang et al. reported that heroin added to human macrophage cultures inhibited both IFN-α and IFN-β, which are antiviral molecules (67). Fecho et al. using slow-release morphine pellets in rats showed that spleen cells placed *ex vivo* were inhibited in responses to ConA (73, 74). They concluded that the immunosuppression occurred by production of nitric oxide. This result is somewhat anomalous as nitric oxide is considered a product of activated macrophages, and, as documented above, morphine pellets result in macrophages that are not activated, but are down-regulated as determined by depressed production of pro-inflammatory cytokines. In support of the Fecho observations, Khabbazi et al. (75) found that morphine blocked the transition of murine primary bone marrow macrophages and the RAW264.7 cell line to the alternatively activated state M2 state induced by IL-4. This inhibition occurred by blocking IL-4 mediated induction of matrix metallopeptidase 9 (MMP-9) and arginase-1, markers of M2 macrophages. Since arginase-1 inhibits nitric oxide production associated with M1 macrophages, the effect of morphine would be to favor this activation pathway. Findings from the Borea laboratory support a role for morphine in amplifying LPS-induced activation of primary murine microglia (76, 77). This group activated the microglia *in vitro* with LPS, and found that subsequent exposure to morphine increased the LPS-mediated production of IL-1β, TNF-α, IL-6 and nitric oxide, and this occurred via activation of PKCε and the Akt pathway upstream of ERK1/2 and inducible nitric oxide synthase (76). Further, low doses of morphine activated NF-κB via PKCε (77). However, morphine alone (without LPS) has no effect in either assay. Other investigators have shown that an incision in the paw of a rat (78) or a mouse (79) induced pro-inflammatory cytokines whose production was unchanged or depressed by morphine. Limiroli et al. reported that acute doses of morphine *in vivo* suppressed both IL-12 and IL-10 in thioglycolate-elicited peritoneal macrophages placed in culture and stimulated with LPS, with or without IFN-γ (80). The Sacerdote laboratory also found that peritoneal macrophages obtained 1 h after a single, s.c. injection of morphine had reduced levels of IL-1β, TNF-α, and IL-12 in response to stimulation with LPS (81). Long et al. reported that addition of morphine to human monocytes in culture depressed levels of TNF-α, and increased the anti-inflammatory cytokine, IL-10 (82). An additional complication is the observation by Roy et al. that micromolar doses of morphine added to murine peritoneal macrophages inhibited IL-6 and TNF-α, whereas nanomolar doses of the opioid up-regulated these pro-inflammatory cytokines (83). These opposite dose-dependent results correlated with depressed and activated levels of NF-kB. Infection of mice with *Acinetobacter baumannii* or *Streptococcus pneumoniae* has been shown to induce the cytokines IL-17 (produced by T cells) and IL-23 (produced by macrophages and dendritic cells). Mice implanted with slow-release morphine pellets have depressed levels of these pro-inflammatory cytokines, which correlates with increased susceptibility to these infections (84–86). Other studies have examined production of cytokines produced by T cells. Jessop and Taplits reported that morphine added to mouse spleen cells stimulated with ConA *in vitro* had reduced production of IL-2 and IL-4 (87). Similarly, Roy et al. showed that mouse thymocytes placed in culture and stimulated with PHA and IL-1β had a dose-dependent reduction in secretion of IL-2 when treated with morphine, that correlated with down-regulation of the transcription activator *fos* (88). Lysle et al. administered morphine by s.c. injection to rats and showed a dose-dependent suppression of production of IL-2 and IFN-γ by splenocytes placed *ex vivo* and stimulated with ConA (89). Almost all of these studies showed that morphine down-regulates cytokine production, both cytokines that are produced by macrophages and those that are produced by T cells.

## Opioids, Cell Movement, Chemokines, and Chemokine Receptors

### Effects of Opioids on Cell Movement

Morphine slow-release pellets have been shown to depress leukocyte sticking and rolling along blood vessels in response to oxidized low-density lipoprotein, as visualized using intravital fluorescence microscopy of cells in dorsal skin-fold windows implanted in mice (90). Such inhibition is anti-inflammatory. In studies examining the anti-tumor effects of morphine, serum taken from mice treated with morphine was found to inhibit *in vitro* migration of bovine aortic endothelial cells and mouse mammary breast carcinoma cells, and to reduce the *in vitro* invasion capacity of these tumor cells (91). These effects were attributed to decreased serum levels of metalloproteinase 9 (MMP-9) and increased levels of tissue inhibitor of metalloproteinases 1 and 3/4. In a further study by this group, morphine was shown to inhibit IL-4 driven differentiation of macrophages into the M2 state, which correlated with reduced levels of MMP-9 (75). As the M2 state is usually associated with tumor progression, theses studies provide a mechanism by which morphine can exert an anti-tumor effect. Morphine has also been shown to suppress growth of Lewis Lung Carcinoma cells in muce that correlated with reduced production of VEGF (vascular endothelial growth factor) and reduced blood vessel density, length and branching (92). The effect was found to be due to suppression of the p38 MAPK pathway which inhibited VEGF transcription and secretion. Morphine has also been shown to delay wound healing by inhibiting VEGF synthesis as well as recruitment of neutrophils and monocytes to the site of injury (93).

### Effects of Opioids on Chemotaxis and Levels of Cytokines and Chemokines

A much larger literature exists describing effects of morphine added *in vitro* to chemotaxis by phagocytic cells in the periphery, and microglia and astrocytes in the CNS. Chao et al. reported that morphine inhibited primary human fetal microglial cells from chemotaxis in response to C5a, with an IC_50_ value of 1 fM (94). The inhibition was blocked by the opioid antagonist, β-funaltrexamine (β-FNA). Mahajan et al. found that addition of morphine *in vitro* to the astrocytoma cell line U87 or to primary human astrocytes resulted in a dose-dependent reduction in mRNA and protein production of the chemokines CXCL1/IL-8/KC, CCL4/MIP-1β, and CCL2/MCP-1 (95, 96). Similarly, the Peterson laboratory found that *in vitro* treatment of human microglial cultures with morphine inhibited production of CCL5/RANTES in response to LPS and IL-1β (97). In both of these studies, β-FNA, the MOR antagonist, reversed the inhibitory effect of morphine. The Hauser laboratory reported that morphine added to primary mouse astrocytes or microglia, or a microglial cell line (N9), did not induce pro-inflammatory cytokines or chemokines (IL-6, G-CSF, CCL5/RANTES, CCL2/MCP-1, or CCL12/MCP-5) (98, 99). Interestingly, this group also showed that morphine itself had weak chemotactic activity for the N9 mouse microglial cell line (100). Conditioned media from primary mouse astrocytes exposed to morphine also slightly enhanced chemotaxis of N9 cells (101). The magnitude of stimulation of microglial activation by morphine alone was small compared to the effect on chemotaxis observed when morphine was combined with the HIV protein, Tat (101). In an experiment to test the *in vivo* effects of combining morphine and Tat on numbers of astroglia and macrophages, mice were implanted with morphine pellets and Tat given into the striatum. Treatment with both Tat and morphine resulted in increased cellularity, but morphine alone had no effect, so the opioid by itself was not found to up-regulate a cellular inflammatory response in this area of the brain (101). Dutta et al. also investigated the effect of morphine on primary mouse microglia in combination with Tat and *Streptococcus pneumoniae*. They also found that neither morphine or Tat alone resulted in production of TNF-α, IL-6 or MCP-1/CCL2, but the combination of all three stimulators synergized to produce a 4-fold increase in these cytokines (102). Another laboratory has shown that morphine induces the chemokine IP-10 in a human astroglial cell line, A172, and that the effect is blocked by β-FNA (103). In contrast to the results of Mahajan et al., Chao et al., and Hu et al. using morphine, DAMGO, a synthetic compound that is a selective agonist for the MOR, when added to human PBMCs (in contrast to astrocytes or microglia) that were stimulated with PHA, was found to up-regulate CCL2/MCP-1, CCL5/RANTES, and also interferon-inducible protein-10/IP-10 (104). Chao et al. had also used DAMGO to test its effects on primary fetal microglial and found that, like morphine, it inhibited the chemotactic response to C5a. At present, there is no explanation for why there are opposite effects of DAMGO reported by different laboratories, except to note that different types of cells were used in the different experiments. A prior review of the literature on opioid-mediated immunosuppression, that included effects of peptide agonists at opioid receptors (105), found that, in contrast to morphine, which was uniformly immunosuppressive, endogenous peptide agonists were frequently immunostimulatory. The reader may recall that the very first paper cited in this review by Wybran et al. found that morphine depressed T cell rosetting with SRBCs, but me-enkephalin enhanced resetting (1). Makman et al. also reported that morphine, but not peptide opioids, blocked the activation of human granulocytes by TNF-α and chemotaxis induced by CXCL1/IL-8 (106). It is notable that DAMGO is a peptide that is an analog of leu- and met-enkephalin with selectivity for the MOR, so perhaps its effects on the immune system are more similar to those of the endogenous opioid ligands. There is strong evidence that differences between activity of opioid peptides and alkaloids can be attributed to agonist-biased signaling or functional selectivity (107–110). Other studies have assessed the effects of morphine on chemokine receptor expression. Mahajan et al. found that morphine added to astrocyte cell lines up-regulated CCR5, CCR3, and CXCR4 levels in the face of down-regulating production of certain chemokines (see above) (95). Guo et al. reported that morphine increased CCR5 in human monocyte-derived macrophages (111), and Suzuki et al. showed a similar increase in CCR5 by methadone in a human lymphocytic cell line (112). Steele et al. reported that DAMGO induced increased expression of CCR5 and CXCR4 in human monocytes and T cells, effects which were blocked by both naloxone and the MOR-selective antagonist, CTAP (66). Overall, morphine appears to up-regulate chemokine receptor expression, while down-regulating chemokine levels. Perhaps the decrease in chemokine levels is due to their more avid uptake by the increased number of receptors. The papers investigating the effects of opioids on chemokines and chemokine receptors are tabulated in Table 2.

**Table 2:** Opioid effects on chemokine levels.

**Chemokine ligands**	**Species**	**Opioid**	**Effect**	**References**
CCL2/MCP-1	Human *in vitro*	Morphine	↓	(95, 96)
	Mouse *in vitro*	Morphine	↓	(98, 99)
	Human *in vitro*	DAMGO	↑	(104)
CCL4/MIP-1β	Human *in vitro*	Morphine	↓	(95, 96)
CCL5/RANTES	Human *in vitro*	Morphine	↓	(97)
	Mouse *in vitro*	Morphine	↓	(98, 99)
	Human *in vitro*	DAMGO	↑	(104)
CCL12/MCP-5	Mouse *in vitro*	Morphine	↓	(98, 99)
CXCL1/IL-8/KC	Human *in vitro*	Morphine	↓	(95, 96)
CXCL10/IP-10	Human *in vitro*	Morphine	↑	(103)
	Human *in vitro*	DAMGO	↑	(104)
**Chemokine receptors**				
CCR3	Human *in vitro*	Morphine	↑	(95)
CCR5	Human *in vitro*	Morphine	↑	(95, 111)
	Human *in vitro*	Methadone	↑	(112)
	Human *in vitro*	DAMGO	↑	(66)
CXCR4	Human *in vitro*	Morphine	↑	(95)
	Human *in vitro*	DAMGO	↑	(66)

### Heterologous Desensitization Between Opioids and Chemokine Receptors

Another aspect of the intersection of opioids with chemokines relates to interactions between their receptors. Both opioid and chemokine receptors belong to the class of G protein-coupled seven transmembrane receptors (GPCRs). There is a body of work showing that these two sub-types of GPCRs can cross-desensitize each other. Thus, when an opioid receptor binds its ligand, it blocks a chemokine receptor from signaling when it binds its chemokine ligand. As will be documented below, this heterologous desensitization is bi-directional, as chemokines binding to chemokine receptors can block opioid receptors from signaling. Makman et al. showed that human neutrophil chemotaxis to fMLP, a peptide chemotactic factor was inhibited by morphine (106). Miyagi et al. found that morphine blocked chemotaxis of monkey neutrophils and monocytes to CXCL1/IL-8 and CCL5/RANTES in a dose-dependent manner that was naloxone inhibitable (113). The Oppenheim laboratory reported that met-enkephalin inhibited the chemotaxis of human neutrophils in response to CXCL1/IL-8, and of human primary monocytes to CCL3/MIP-1a or CCL2/MCP-1 (114). Further studies showed that HEK293 cells which were transfected to express MOR and CCR1, when pretreated with an opioid, failed to respond to the chemokine, CCL3, a CCR1 ligand (115). The Adler laboratory made the seminal observation that administering either CXCL12/SDF-1α or CCL5/RANTES into the periaqueductal gray (PAG) region of the rat brain blocked the analgesic effect of the mu-selective opioid, DAMGO, given 30 min later into the PAG, as measured by the cold-water tail flick assay (116). Further studies showed similar results using morphine (117). These novel results led to the conclusion that chemokines inhibit morphine analgesia. This pathway was explored further by showing a direct effect of chemokines on neuronal transmission in electrophysiological studies of PAG neurons which showed that the chemokines, CXCL12 and CX3CL1 inhibited morphine-induced hyperpolarization and reduction of input resistance (118). The Adler group has also shown that the chemokine receptor antagonists, AMD3100 (an antagonist at CXCR4) and maraviroc (an antagonist at CCR5), when used in combination with sub-analgesic doses of morphine in an incisional pain assay in rats, shifted the morphine dose-response curve 3.3-fold to the left (78). Similar effects were observed with oxycodone and meperidine (119). The heterologous desensitization between CXCL12 and an opioid has been confirmed by Rivat et al. who reported that this chemokine blocked morphine-mediated analgesia in a rat paw pressure pain model, and a different CXCR4 antagonist blocked the inhibitory effect of the chemokine and enhanced morphine analgesia (120). These robust results on chemokine-opioid interactions have led to the synthesis of a bivalent compound that has activity at both the mu receptor and the CCR5 receptor. It has been shown to block inflammatory and neuropathic pain in mice in response to bacterial lipopolysaccharide (121) and to arthritis (122). Padi et al. has tested a synthetic dual antagonist (RAP-103) that binds to both CCR2 and CCR5, and shown that it has analgesic activity without use of an opioid in a rat model of chronic constrictive nerve injury (123). The potential of exploiting the interactions between chemokines and opioids for pain therapy has been recently reviewed in greater detail (124). The biological basis for the heterologous desensitization phenomenon has been investigated. Grimm et al. first reported that phosphorylation of the intracellular signaling tail of the CXCR1 and CXCR2 chemokine receptors by the opioid blocked the ability of the chemokine receptor to signal (114). There is also considerable evidence using transfected cell lines that chemokine receptors can form heterodimers with opioid receptors that can co-immunoprecipitate (125, 126). *In vivo* immunohistochemical evidence also supports the possibility that these two receptor types can interact, as single neurons in the PAG were shown to express both MORs and CXCR4 receptors (118), as were neurons in the dorsal root ganglion and spinal cord (120). In novel studies, Rivat et al. have shown that CXCL12 activates Src family kinases (SFK) *in vitro* in primary cultured lumbar rat dorsal root ganglia and *in vivo* in the DRG and spinal cord. An SFK inhibitor blocked CXCL12-induced depression of morphine analgesia, providing evidence that SFKs mediate chemokine effects on opioid receptors. Rogers has carried out studies on mechanism of heterologous desensitization of the CCR5 receptor by DAMGO on the chemotactic response of human primary monocyte-derived macrophages and transfected cell lines, and found that it involves phosphorylation of CCR5 by protein kinase C ζ (PKC ζ) (127). Major conclusions from this body of work are that morphine can dampen inflammation by blocking chemotaxis of inflammatory cells, and that inflammation can block opioid analgesia by releasing chemokines that desensitize opioid receptors.

## Molecular Mechanisms of Opioid-Mediated Immunosuppression

As this field has progressed, investigators have dissected the mechanisms of opioid-induced immunosuppression. In Roy's laboratory, it was shown that murine peritoneal macrophages treated with micromolar doses of morphine had depressed NF-κB levels, but nanomolar doses of morphine led to NF-κB activation (83). In mice given morphine pellets, there was inhibition of activation of NF-kB-dependent gene transcription in alveolar macrophages infected with the bacterium, *Streptococcus pneumoniae*. Martucci et al. used RelB k/o mice to probe further the role of NF-κB in morphine-mediated immunosuppression (81). An acute s.c. injection of morphine was administered to WT or RelB k/o mice and peritoneal macrophages from the two mouse strains were harvested and stimulated in culture with bacterial LPS. IL-1β, TNF-α, and IL-12 were reduced in morphine-treated cells from WT, but not RelB k/o mice. Nitric oxide production was also ablated by morphine in WT, but not RelB k/o mice. In splenocytes of morphine treated mice, IL-2 and IFN-γ were reduced in WT but not k/o animals. These studies corroborate the findings cited above showing that morphine mainly suppresses pro-inflammatory cytokines and provide a molecular pathway by which this immunosuppression occurs via suppression of NF-κB. Ho's laboratory explored the mechanism by which heroin enhanced HIV replication in human macrophages in culture by inhibiting a group of miRNAs (miRNA-28, miRNA-125b, miRNA-150, and miRNA-382) that restrict HIV replication (67). Long et al. pursued the molecular mechanisms by which morphine and heroin are immunosuppressive. Using an miRNA array they found that the levels of miRNA-582-5p and miRNA-590-5p were depressed in PBMCs of heroin abusers (82). Further, when primary human monocytes were treated with morphine there was a concentration dependent suppression of these miRNAs and, in agreement with Wang et al., down-regulation of NF-kB (128). Transfection of human monocytes with mimics of these two miRNAs increased TNF-α and decreased IL-10 production, and increased NK-κB levels. This group also showed that the morphine-induced immunosuppression is mediated by the miRNAs binding to CREB1/CREB5. Li et al. examined the role of miRNAs in morphine-induced macrophage apoptosis. They found that morphine depresses miR-873 in murine peritoneal macrophages and spleen. Apoptosis of cells from the morphine-treated groups could be inhibited by treatment with miR-873 mimics (129).

## Opioids and Potentiation of Infection and Sepsis

The literature reviewed above shows that, with few exceptions, opioids are immunosuppressive. It is well-recognized in the clinical literature that opioid addicts have increased rates of infection, and the intersection of HIV infection with intravenous drug abuse is well-established (130–135). There are also studies showing increased infection rates, particularly for pneumonia, in patients who were not abusing opioids, but receiving them long-term for treatment of pain (136–138). As reviewed by Plein and Rittner (139), there is also a contradictory study by W. Hauser et al. (140) that did not find a correlation of increased infection with opioid use. In clinical studies there are many confounding factors. It is useful, therefore, to examine the preclinical literature investigating the effects of opioids on a variety of infections where variables can be precisely controlled. The results of studies of opioids on infection are shown in Table 3. Among the first reports on this subject in 1983 was that of Tubaro et al. which showed that morphine sensitized mice to infection with the fungus, *Candida albicans*, and to the Gram negative bacterium, *Klebsiella pneumonia* (141). Mice injected with morphine at 3, 24, and 48 h after Klebsiella infection had a 3-fold reduction in the LD_50_ compared to vehicle controls. Animals challenged with Candida were given morphine either by osmotic pump or by two different dosing regimens on days after infection. It was found that morphine, compared to vehicle, markedly decreased the number of surviving mice and halved the mean time to death. As mentioned earlier in the review, this group also showed that mechanisms by which morphine potentiated these infections was to depress phagocyte recruitment and microbicidal activity of phagocytes. As noted above, morphine enhanced HIV replication in monocytic cells (65, 66, 68, 142). The K. Hauser laboratory has also shown that morphine increases the cytotoxicity of HIV Tat by up-regulating pro-inflammatory cytokines when the two molecules are combined (98, 100). In contrast, morphine attenuated Friend leukemia virus, a murine retrovirus, *in vivo* (143). However, the opioid potentiated the development of herpes simplex virus, type 1 encephalitis in mice when a single dose of the drug was given prior to infection (144, 145). In regard to bacteria, the Eisenstein laboratory has shown that morphine pellets dramatically sensitized to oral infection of mice with *Salmonella typhimurium* (146). Control mice given a dose of Salmonella that produced 50% survival, had a mean survival time of 28 days, whereas mice given morphine pellets in doses ranging from 16 to 75 mg all died by day 5. This dramatic potentiation of Salmonella infection by morphine was confirmed by counts of Salmonella in the Peyer's Patches, mesenteric lymph nodes and spleen. No organisms were found on the first to third days after inoculation in naltrexone treated mice, but morphine treated mice had bacterial burdens as high as 10^6^ per tissue analyzed. An anomalous finding was that mice that received a morphine pellet and a naltrexone pellet had prolonged mean time to death compared to those receiving only morphine, but 100% of the animals still succumbed to the infection. To prove that the enhanced sensitivity to Salmonella infection induced by morphine was mediated by the MOR, MOR k/o mice were used. It was shown that when MOR k/o mice given morphine pellets were compared to wild-type animals, the former were resistant to doses of Salmonella that were lethal for the normal mice. Further, when WT and MOR k/o mice were treated with morphine and given the same oral dose of Salmonella, the bacterium was cultured from the blood and peritoneal fluid of the WT, but not the k/o mice. This study demonstrated that the morphine-mediated enhancement of oral Salmonella infection was dependent on the presence of the MOR (84). Interestingly, animals became tolerant to the opioid-sensitizing effect on Salmonella infection by 96 h after morphine pellet implantation (146). However, if the pellets were removed, which put the animals into withdrawal, they were again sensitized to oral Salmonella, as well as to systemic infection with this organism (147, 148). Morphine pellets or osmotic pumps are common ways to deliver a continuous dose of morphine so that animals do not have to be injected several times a day with the opioid. It is worth noting that morphine delivered by osmotic pump was less sensitizing to Salmonella infection than pellet implantation (147). Roy's laboratory showed that morphine pellets sensitized to intranasal infection of mice with another bacterium, *Streptococcus pneumoniae* (61, 128). Increased susceptibility to this infection correlated with delayed neutrophil recruitment which correlated with lower levels of the chemokines CXCL1/KC/IL-8, CXCL2/MIP-2 and depressed levels of TNF-α, IL-1, IL-6, and IL-17/IL23 in bronchoalveolar lavage fluid (61, 85). The effect of morphine on pneumococcal infection in the mice was mediated by the MOR, as MOR k/o mice were not sensitized to this infection by morphine (102). Morphine added to alveolar macrophages from normal mice inhibited their ability to respond to *in vitro* infection with the pneumococci with production of CXCL2/MIP-2 (128). Morphine also down-regulated NF-κB which correlated with the decrease in pro-inflammatory cytokines (128). These investigators concluded that morphine compromised the innate immune responses to this pathogen. Breslow et al. showed that morphine pellets sensitized to infection with *Acinetobacter baumannii*, as evidenced by increased mortality for the same challenge dose of bacteria and by higher Acinetobacter burdens in blood, lungs, livers and spleens of mice given the opioid compared to placebo-treated animals (149). Pro-inflammatory cytokines (IL-6, TNF-α, IL-12, IFN-γ, and the chemokine CCL2/MCP-1) were up-regulated in plasma of morphine-treated mice collected 8 h after infection, which was interpreted to be the result of the increased systemic bacterial burdens with the pathogen in animals given morphine. In peritoneal exudate fluid IL-17A and the chemokine CXCL1/KC/IL-8 were depressed. Depression in these two chemotactic molecules correlated with depressed accumulation of inflammatory cells in the peritoneal cavity in morphine-treated animals. Asakura et al. reported that morphine dramatically sensitized mice to infection with *Listeria monocytogenes* (150). Animals were given a sub-lethal dose of the bacteria which gave 100% survival in controls, but 100% mortality in morphine-treated mice. An early study from the Peterson laboratory (151) found that mice given a single injection or multiple injections of morphine over a period of several days led to an addicted status, and they were susceptible to a sublethal dose of the parasite, *Toxoplasma gondii*. Morphine treated animals suffered 86% mortality, while there was no mortality in control animals. When the Toxoplasma was given 9 or more days prior to giving morphine, a similar potentiation of mortality was observed. Naloxone blocked these effects. Reports from Singh et al. and Singal et al. found that low doses of morphine suppressed infection of mice with *Plasmodium berghei* and hamsters with *Leishmania donovani*, whereas high doses of morphine sensitized the animals to these parasitic infections (152–154). A study which did not fit the pattern was that of Singh et al. who found that morphine inhibited growth of *Mycobacterium tuberculosis* in mice (155). This observation is at odds with the clinical observation that people who take opioids are at greater risk for infection with Mycobacteria (156). Among the most interesting observations made concerning opioids and infection relates to effects of the drug on GI transit. A well-known side effect of opioid administration is inhibition of gastric transit (157). In 1997, Hilburger et al. reported that morphine pellet implantation led to sepsis in mice (158). Sepsis was defined as presence of organisms that were part of the normal flora of the mouse intestine in the mesenteric lymph node, peritoneal cavity, spleen and liver of morphine pelleted mice. Organisms that were detected included *Proteus mirabilis, Enterococcus faecalis, and Eshcerichia coli*. Further, morphine sensitized mice to endotoxic shock induced by injection of lipopolysaccharide. It was hypothesized that morphine compromised the gut epithelial barrier which resulted in leakage of organisms into the systemic circulation. Interestingly, Holaday's laboratory had shown years earlier that rats and dogs could be protected from endotoxin-induced hypotensive shock by treatment with naloxone, suggesting that endogenous opioids might be involved in this syndrome (159, 160). As hypotensive shock frequently accompanies bacterial sepsis, and shock is a major complication leading to fatality from Gram negative bacterial sepsis, the involvement of opioids in both phenomena is notable. Other researchers confirmed the Hilburger observation (161, 162). Roy's laboratory extended these findings into the mechanism by which morphine opens the epithelial barrier (163). They showed by immunohistochemistry that morphine pellets induced disruption of tight junction organization in the ileal, but not the colonic mucosa, which led to leakage of bacteria from the intestinal tract. Although the ileum contains a smaller burden of normal flora than the colon, they did confirmatory experiments and showed that normal commensal bacteria were found in the mesenteric lymph nodes and livers of morphine-treated mice. Further, ampicillin-resistant *Eshcerichia coli*, introduced by gavage, similarly translocated to the systemic compartment in morphine treated mice. TLR4 was involved in this effect, as TLR4 k/o mice did not respond to morphine with increased gut permeability. Additional observations from the Eisenstein group are that when mice were put into withdrawal from morphine by removing the pellets, they again became septic, meaning presence of normal gastrointestinal flora in the systemic compartment, and were sensitized to oral and to intraperitoneal infection with Salmonella (148, 164). Sepsis occurring during withdrawal was documented by detection of *Enterococcus faecium* and *Klebsiella pneumoniae* in mesenteric lymph nodes, spleens, livers, and peritoneal cavities of withdrawn animals. Withdrawal also correlated with increased levels of pro-inflammatory cytokines and a chemokine, including IFNγ, TNF-α, IL-6, and CCL2/MCP-1, and depression of IL-10 in animals infected with Salmonella or given LPS (164, 165). Splenic nitric oxide was also elevated in withdrawn animals given LPS (165). Withdrawn animals were highly sensitized to mortality induced by bacterial LPS, but anti-TNF-α antibody was protective (165). This constellation of pro-inflammatory mediators is consonant with what is observed in humans that are septic or undergoing hypotensive shock. A surprising but unexplained observation was that in opioid withdrawn animals infected with Salmonella or given LPS, serum, and plasma IL-12 was depressed, as was splenic mRNA for IL-12, which occurred in the face of increases in the other pro-inflammatory markers (164, 165). In summary, morphine has mainly a potentiating effect on microbial models of infection in laboratory animals, which correlates with immunosuppression (Table 3). However, an underlying, and potentially overlooked consequence of morphine administration and withdrawal is the possibility that lipopolysaccharide may be released into the circulation from escape of normal Gram negative flora from the GI tract, which would be pro-inflammatory. As some protocols for morphine administration by injection may put animals into periods of withdrawal between injections, sepsis might occur, and release of inflammatory cytokines might result. Investigators might erroneously conclude that morphine is directly augmenting inflammation, when the inflammation may be secondary to underlying sepsis that has gone undetected.

**Table 3:** Opioids and sensitization to infections.

**Organism**	**Species**	**Effect**	**References**
*Acinetobacter baumannii*	Mouse *in vivo*	Potentiation	(149)
*Candida albicans*	Mouse *in vivo*	Potentiation	(141)
Friend leukemia virus	Mouse *in vivo*	Attenuation	(143)
Herpes simples virus, type 1	Mouse *in vivo*	Potentiation	(144, 145)
HIV	Human *in vitro*	Potentiation	(65–68, 142)
*Klebsiella pneumoniae*	Mouse *in vivo*	Potentiation	(141)
*Leishmania donovani*	Hamster *in vivo*	Low dose: attenuation High dose:potentiation	(153)
*Listeria monocytogenes*	Mouse *in vivo*	Potentiation	(150)
*Mycobacterium tuberculosis*	Mouse *in vivo*	Attenuation	(155)
*Plasmodium berghei*	Mouse *in vivo*	Low dose: attenuation High dose:potentiation	(152)
*Salmonella typhimurium*	Mouse *in vivo*	Potentiation	(84, 146–148)
*Streptococcus pneumoniae*	Mouse *in vivo*	Potentiation	(61, 85, 102, 128)
*Toxoplasma gondii*	Mouse *in vivo*	Potentiation	(151)

## Are Opioids Pro-inflammatory via Triggering of Toll-Like Receptor 4 (TLR4)?

### Do Opioids Mediate Opioid Dependence by Being Pro-inflammatory?

The reader must appreciate from the literature cited above, that almost all of the studies carried out on effects of morphine and heroin on the immune system have concluded that opioids are immunosuppressive. Starting in the last decade, an unexpected hypothesis has emerged, namely, that morphine activates glia, and that glial activation via release of pro-inflammatory cytokines mediates morphine tolerance (166). While there is a clear literature linking pain to up-regulation of pro-inflammatory cytokines and chemokines [reviewed in (166, 167)], there has been scant evidence linking morphine to immune activation as illustrated by the extensive literatu**re** cited above. Hutchinson et al. showed that morphine given intrathecally (i.t.) to rats resulted in analgesia to a painful radiant heat stimulus (167). The effect of morphine was transient, dissipating after approximately 100 min. Blocking TNF-α with TNF soluble receptor, blocking IL-6 with neutralizing antibody, or blocking nitric oxide with an inhibitor, all prevented the loss of morphine analgesia over another 100 min, or restored the analgesic effect of the opioid. Minocycline, a putative inhibitor of microglial activation, also prolonged morphine analgesia. Further, morphine administered i.t. for 7 days resulted in up-regulation of mRNA for IL-1β, IL-6, and TNF-α in lumbar dorsal spinal cord tissue. In cerebrospinal fluid, protein levels of several cytokines and chemokines were up-regulated by this chronic i.t. treatment with morphine. Authors concluded from these studies that morphine induced the cytokines. Among the observations was that this chronic i.t. morphine up-regulated mRNA for the pattern recognition receptor, TLR4. This observation was pursued by demonstrating that antagonists of TLR4, an *E. coli* mutant LPS and LPS from *Rhodobacter sphaeroides*, were also able to prolong analgesia induced by i.t. morphine (168). When morphine analgesia was assessed in TLR4 k/o animals, the morphine dose-response curve was shifted 3-fold to the left, indicating that TLR4 stimulation was inhibiting morphine's capacity to induce analgesia (168). *In vitro* experiments used HEK293 cells transfected to express human TLR4. These cells are negative for the MOR. Both the active (–) and inactive (+) stereoisomers of morphine, as well as oxycodone, methadone, buprenorphine, and fentanyl all induced TLR activation as measured by a reporter system using fluorescently labeled alkaline phosphatase (168, 169). The doses of morphine used were 10–100 μM, which are high, and generally considered non-physiological for immune cells. Immunosuppressive effects of morphine *in vitro* are observed in other assays in the range of 1–0.001 μM (43). Other novel observations were that both (+) and (–) naloxone were active in blocking many of these effects (170, 171). The Watkins group has also published several other provocative papers providing more direct evidence that morphine can interact with TLR4. They carried out an enzyme-linked immunosorbent assay (ELISA) in which biotinylated morphine was used to attach to 96 well plates coated with streptavidin. Protein A-tagged MD-2, a molecule needed to complex with TLR4 for the receptor to signal, was added to the morphine coated plates. MD-2- morphine complexes were detected by adding an IgG-horseradish peroxidase conjugate (which recognizes the Protein A). It was found that MD-2 bound in a concentration dependent manner to morphine. When MD-2 was immobilized on the plate, morphine was shown to bind to it, and LPS inhibited the binding, suggesting that morphine and LPS compete for binding to MD-2 (169). Other experiments showed that morphine added to the TLR4 and MD-2 transfected HEK cells caused TLR4 oligomerization and co-precipitation of morphine with MD-2 (169). X-ray crystallographic molecular modeling analysis provided evidence that morphine could induce conformational changes in MD-2 similar to those induced by LPS. Experiments were also carried out using primary human CNS endothelial cells *in vitro*, to which the (+) enantiomer of morphine was added (which is inactive at the MOR). At 100 μM but not at 10 or 1 μM, (+) morphine induced mRNA for IL-1β. Again, this is a high dose of morphine. It is unclear why these studies were carried out with endothelial cells, since the cells of choice would be microglia or macrophages, both of which express much higher levels of TLR4 than endothelial cells. Additional work from this group showed that morphine added to a rat microglial cell line, HAP1 cells, induced COX-1 mRNA, which was inhibited by minocycline (172). Here too, the doses of morphine were 100 and 1,000 μM, which in most *in vitro* immune assays are considered potentially toxic. These observations have been extended to evaluate the role of TLR4 in opioid drug reinforcement. Both TLR4 and MyD88 (a signal transducing molecule for TLR4) k/o mice showed decreased conditioned place preference in response to morphine (173). The inactive enantiomer of naloxone (+), which was demonstrated to have activity at TLR4, blocked remifentanil self-administration in rats (173). In a model of neuropathic pain induced by dorsal root avulsion, morphine for 7 days resulted in development of mechanical allodynia. If (+) naloxone was co-administered with morphine, mechanical allodynia did not develop. As their other studies showed that (+) naloxone was an antagonist at TLR4, the interpretation of this experiment was that the morphine-induced hyperalgesia was occurring through TLR4 signaling (174). In aggregate, the body of work from this group has led them to the conclusion that morphine and other opioids induce inflammation by binding to TLR4, and that the inflammatory response mediates pain and also behaviors associated with addiction (175, 176). Roekel et al. (177) tested the hypothesis that a receptor other than MOR could be involved in morphine-induced hyperalgesia by using MOR k/o mice. They found that repeated morphine injections led to analgesic tolerance and hyperalgesia only in WT but not mu k/o animals, showing that only the mu receptor is needed to mediate these biological effects. This group also found that the morphine metabolite, morphine-3-glucuronide (M3G), elicited hyperalgesia in WT but not MOR k/o mice, a result which abrogates the need for another receptor like TLR4 in these processes. In contrast, studies from the laboratory of White (178) support the observations of the Watkins group. They found that M3G, which is devoid of analgesic activity but induces hyperalgesia, displayed this activity in WT mice, but not in TLR k/o mice. In addition, either LPS or M3G induced excitatory changes in neurons in the dorsal root ganglion that were blocked by an inhibitor of the TLR4/MD-2 complex. Stevens et al. found contradictory results to those of the Watson group. They reported that morphine inhibited LPS stimulation of a reporter cell line in which the hTLR4 receptor was transfected into HEK-Blue cells (179). The Parat laboratory tested the ability of morphine, M3G and M6G to activate these same cells and found that neither morphine nor M6G signaled through TLR4, but M3G had weak activity (180). Further serum of morphine treated mice had NF-kB activating activity that was TLR4 and M3G independent. Interestingly, morphine and M3G could block LPS activation of TLR4, a finding that is in agreement with the literature supporting the conclusion that morphine is immunosuppressive. The DeLeo laboratory (181, 182) observed that morphine given chronically i.t. resulted in microglial activation, and that blocking glial activation with propentofylline reinstated the effectiveness of acute morphine (182). However, the role of TLR4 in microglial activation by morphine was not tested in these studies.

There are some reports that morphine can activate microglia and the hypothesis is being tested that pro-inflammatory cytokines released by these cells may mediate the addiction process or be involved in withdrawal symptoms. In light of the reports from the Watkins laboratory, the question is raised as to whether morphine can activate microglia via either the MOR or the TLR4 receptor? Using transcriptomic analysis and fluorescent reporter mice, the Gaveriaux-Ruff laboratory presented convincing evidence that mouse brain microglia express MOR (183). However, other laboratories did not find MOR expression in spinal microglia (184, 185). The Roy laboratory has reported that primary murine brain microglial cells express mRNA and protein for TLR4, and that morphine treatment *in vivo* results in increases in both mRNA and protein expression of TLR4 in murine and human microglia (102). The MOR antagonist CTOP inhibited this effect and the morphine-induced expression of TLR was abolished in MOR k/o mice. The Sacerdote laboratory added morphine to the RAW264.7 macrophage cell line *in vitro* and found decreased TLR4 at the mRNA and protein levels (186). When morphine was injected into mice, harvested peritoneal macrophages also had decreased TLR4 mRNA and protein. MOR k/o mice did not induce these changes in TLR4, demonstrating that suppression of TLR4 by morphine occurs via the classical MOR. Gessi et al. found that morphine enhanced NF-kB in LPS-pretreated murine microglia, which would be a molecular precursor to microglial production of proinflammatory cytokines (77). However, morphine alone, without the LPS pre-treatment, had no effect on NF-kB levels in the microglia. If microglia express TLR4, and if morphine were inducing inflammation in microglia by binding to this receptor, or to the MOR, an effect of morphine alone would be expected. Merighi et al., also used LPS-stimulated murine microglia, and reported that morphine and DAMGO increased production of pro-inflammatory cytokines and nitric oxide through induction of a signaling pathway involving the MOR and PKCε, AKT, and ERK1/2 (76). Fukagawa et al. made the observation that treatment of mice with minocycline attenuated the development of morphine tolerance (187). As minocycline is used as an inhibitor of microglial activation, the experiment was interpreted to mean that morphine tolerance was mediated by microglial activation, but this was not TLR4 dependent as it occurred in TLR4 k/o mice. Other evidence that morphine could activate microglia was that observation that the opioid resulted in up-regulate mRNA for CD11b, a microglial (and macrophage) surface marker, in the mouse spinal cord.

The thesis, that morphine triggers TLR4, which mediates induction of pro-inflammatory cytokines, is obviously in contradiction to almost all of the previous literature on the effect of morphine on the immune system, which as detailed in this review shows that morphine is immunosuppressive, not immune-activating. There are several papers from other laboratories that have evidence bearing on this question that do not support the hypothesis that morphine works through TLR4. Bussiere et al. tested the efficacy of morphine pellets on immunosuppression of the *ex vivo* PFC response in several mouse strains. Among these were the C3H/HeJ mice, which have a genetic defect in the TLR receptor that prevents the receptor from signaling. These mice are hyporesponders to bacterial LPS and behave similarly to TLR4 k/o mice. Bussiere et al. found that morphine induced immunosuppression in these naturally TLR4 deficient animals that was as strong as that observed in WT mice (33). If morphine were acting by triggering TLR4, C3H/HeJ mice should be refractory to morphine-mediated immunosuppression. Fukagawa et al. tested the ability of C3H/HeJ or TLR4 k/o mice to develop tolerance to repeated morphine injections, and found that both of these mouse strains became tolerant to the opioid, showing that binding of morphine to TLR4 does not mediate these effects (187). The C3H/HeJ mice, and another mouse strain, B10ScNJ, which is a naturally occurring mutant that is missing the gene for TLR4, were tested by Mattioli et al. for traditional pharmacological effects of morphine including tolerance, hyperalgesia and physical dependence (188). They also found that both of these mouse strains, which have mutant or missing TLR4 receptors, responded like WT animals in regard to all of these parameters, showing that these pharmacological effects did not require the TLR4 receptor. They also tested (+) naloxone and found that like the (–) enantiomer, it possessed some activity in these mouse strains, but it could not be through the TLR4 receptors, because this receptor is defective in these animals. A direct test of some of the findings of the Watkins group was reported by Skolnick et al. The plus and minus enantiomers of naloxone and naltrexone were sent coded to three different laboratories. They used the HEK293 cells transfected with hTLR4 to test for ability of the opioid antagonists to block LPS signaling via the TLR4 receptor. The results were negative. Neither the active nor inactive enantiomers inhibited LPS from signaling through TLR4 (189). Watkins et al. published a rebuttal to the Skolnik paper, emphasizing the other lines of evidence from their laboratory that support their conclusion (190). Hutchison and Watkins have also published a separate position paper defending their observations, particularly the concept that opioid addiction is mediated by inflammation (175).

In summary, there is little support from other laboratories for the hypothesis that morphine signals through the TLR4 receptor. However, there is still the possibility that cytokines and chemokines may be involved in addiction pathways. Valentinova et al. reported that morphine withdrawal induces TNF-a in the lateral habenula that is involved in behavioral modulation (191). A more comprehensive coverage of the role of inflammation in addictive processes and the cellular origin of inflammatory mediators that do not involve morphine triggering of TLR4 is beyond the scope of the present review.

### Can the Two Lines of Evidence Be Reconciled?

This review has covered almost the entire literature on opioids and the immune response. If morphine bound to TLR4 and activated it, releasing pro-inflammatory cytokines, then one would predict that a literature would have developed showing that opioids up-regulate the immune system. As TLR4 is abundantly expressed on macrophages and dendritic cells in the periphery, it would be expected that giving morphine systemically would result in immune activation. Also, many experiments were carried out with macrophages that were exposed to morphine *in vitro* and in most cases macrophage phagocytic and microbicidal activity was suppressed, and cytokine production was depressed. As macrophages are the primary cells expressing TLR4, if morphine bound to TLR and activated this receptor, one would have predicted the opposite result. The molecular studies that are cited above also are almost all consistent with morphine-mediated inhibition of transcription factors and induction of miRNAs that down-regulate inflammatory responses. There should be no argument that procedures that induce pain frequently induce inflammation with production of pro-inflammatory cytokines. Blocking these cytokines alleviates pain, which is not surprising. It also makes sense that the analgesic effect of morphine might be mitigated by blocking the cytokines which are augmenting pain signals. Note, that in these experiments (167), morphine was not tested for induction of inflammatory mediators by glia, rather the pain stimulus induced the cytokines. The section above on the heterologous cross-desensitization between opioids and chemokines provides solid evidence that chemokines can block opioid signaling and interfere with the analgesic effect of opioids. Down-regulation of glia with compounds such as minocycline or propentofylline would also be expected to depress production of chemokines, which could alleviate their blockade of analgesia via the MOR. It is noteworthy that the DeLeo laboratory found that chronic, but not acute, morphine administration resulted in glial activation (181, 182). They also noted that when morphine was given chronically it was only injected twice a day which could result in successive periods of drug withdrawal (182). There is a small literature, reviewed by Eisenstein et al. (192), on effects of withdrawal from opioids on the immune system. Her laboratory showed that acute or precipitated withdrawal from morphine pellets in mice was immunosuppressive (193–196). Other investigators have also found that opioid withdrawal results in immunosuppression including Govitrapong, who examined responses of humans withdrawn from heroin and found their responses to ConA were suppressed for up to 2 years (30). As discussed above in the section on morphine and infection, morphine pellet administration induces sepsis that is detectable 24 and 48 h after pellet implantation (158). Withdrawal from morphine pellets also results in sepsis (148). Sepsis results in leakage of Gram negative organisms and LPS from the gastrointestinal tract. A plausible explanation for how chronic morphine leads to glial activation is that the leaked LPS binds to TLR4 and up-regulates pro-inflammatory cytokine production. The increase in IL-12 and TNF-α observed in peritoneal cells harvested from morphine-pelleted mice and stimulated *in vitro* with LPS plus IFN-γ was attributed to priming of these cells by possible underlying sepsis (197). Leaked LPS may be a cause of morphine-induced allodynia. In regard to the *in vitro* experiments carried out by the Watkins group showing that either enantiomer of morphine binds to MD-2 and triggers LPS with resultant induction of pro-inflammatory cytokines, the possibility that there was LPS contamination must be considered. In their papers, this group does say that they used pyrogen-free saline and water, and endotoxin-free morphine, but they used very high doses of morphine. LPS is active at TLR4 in pg or lower amounts. The fact that inhibitors of TLR4 blocked the effects of morphine would fit with a model in which the morphine contained tiny amounts of LPS, which is ubiquitous and difficult to remove. To prove that effects of compounds which mimic LPS are not due to LPS contamination, investigators frequently see if addition of polymyxin B abolishes the effect. Polymyxin B is highly cationic and binds to the negatively charged LPS, neutralizing it. It would be interesting to see the effect of this drug on some of the observations of the Watson laboratory. In reviewing the literature, one must take into account the findings from studies by other laboratories using mice genetically deficient in the MOR, which is a definitive approach to dissecting the effect of morphine on the immune system. In almost every case, mice lacking the MOR failed to show immunomodulatory effects of morphine. These animals all had intact TLR4 genes and receptors (177). In contrast, mice lacking a functional TLR4 gene still showed morphine mediated immunosuppression (33, 188). If TLR4 were the receptor through which morphine is activating the immune system, one would expect to observe immune activation in most experiments. Clearly, from the extensive review above, that is not the case. Virtually all of the literature shows morphine to be immunosuppressive. Another argument that has been put forth to reconcile the findings from the Watson laboratory with the rest of the literature is that there may be a difference in how morphine acts in the periphery vs. the CNS. In this review, several papers from different laboratories were cited where morphine was added to microglia or astrocytes *in vitro* and mostly immunosuppressive effects were observed (see above under the section on Opioids, Chemokines, and Chemokine Receptors). While beyond the scope of this review, there is a literature showing that neurons produce chemokines and express chemokine receptors. Based on the available evidence, Adler and Rogers have suggested that chemokines may be a third neurotransmitter system in the brain (198). Thus, there is the possibility that various *in vivo* manipulations relating to pain or opioid dependence alter chemokine levels from neurons, and that increased chemokines in this context might not be an indicator of inflammation. One further point is that there is evidence that immunostimulants like cocaine may have pro-inflammatory effects, so one should be careful about generalizing from the opioid literature about the role of chemokines and cytokines mediating effects of other abused substances.

## Summary

This review shows that the vast majority of the literature on the effects of opioids on immune function leads to the conclusion that morphine is immunosuppressive. Most of these experiments followed proper pharmacological design and showed that the immunosuppressive effects were blocked by antagonists at the MOR or by genetic deletion of the MOR. These experiments were carried out in animals and human cells that express TLR4, so if morphine were exerting its effects through the TLR4 receptor, one should have observed immunostimulation and also a failure of MOR antagonists and MOR k/o mice to attenuate the responses. This is clearly not the case. The concept that opioids mediate their pharmacological effects through immune activation via TLR4 is not supported by the published work from many different laboratories.

## Author Contributions

The author confirms being the sole contributor of this work and has approved it for publication.

### Conflict of Interest

The author declares that the research was conducted in the absence of any commercial or financial relationships that could be construed as a potential conflict of interest.
